# Mechanical Properties and Crack Resistance of Basalt Fiber Self-Compacting High Strength Concrete: An Experimental Study

**DOI:** 10.3390/ma16124374

**Published:** 2023-06-14

**Authors:** Zhicheng Xue, Pengfei Qi, Ziran Yan, Qiang Pei, Jintu Zhong, Qinjian Zhan

**Affiliations:** 1 College of Architecture and Engineering, Guangdong University of Petrochemical Technology, Maoming 525000, China; xuezhicheng0630@163.com (Z.X.);; 2 College of Architecture and Engineering, Heilongjiang University of Science and Technology, Harbin 150020, China; 3 College of Architectural Engineering, Dalian University, Dalian 116622, China; q291990@163.com

**Keywords:** basalt fiber self-compacting concrete, orthogonal experiments, mechanical properties, crack resistance

## Abstract

Pure self-compacting concrete has many disadvantages, such as early shrinkage and cracking. The addition of fibers can effectively improve the properties of resistance to tension and cracking of self-compacting concrete, thereby the effect of improving its strength and toughness can be achieved. Basalt fiber is a “new green industrial material” that has unique advantages, such as high crack resistance and being lightweight compared with other fiber materials. In order to study the mechanical properties and crack resistance of basalt fiber self-compacting high-strength concrete intensively, the self-compacting high-strength concrete of C50 was designed and obtained using the absolute volume method with multiple proportions. Orthogonal experimental methods were used to study the influence of the water binder ratio, fiber volume fraction, fiber length, and fly ash content on the mechanical properties of the basalt fiber self-compacting high-strength concrete. Meanwhile, the efficiency coefficient method was used to determine the best experiment plan (water binder ratio 0.3, fiber volume ratio 0.2%, fiber length 12 mm, fly ash content 30%), and the effect of fiber volume fraction and fiber length on the crack resistance of the self-compacting high-performance concrete was investigated using improved plate confinement experiments. The results show that (1) the water binder ratio had the greatest impact on the compressive strength of basalt fiber self-compacting high-strength concrete, and as the fiber volume fraction increased, the splitting tensile strength and flexural strength both increased; (2) there was an optimal value for the effect of the fiber length on the mechanical properties; (3) with the increase in fiber volume fraction, the total crack area of the fiber self-compacting high-strength concrete significantly decreased. When the fiber length increased, the maximum crack width first decreased and then slowly increased. The best crack resistance effect was achieved when the fiber volume fraction was 0.3% and the fiber length was 12 mm. Therefore, basalt fiber self-compacting high-strength concrete can be widely used in engineering fields, such as national defense construction, transportation, and building structure reinforcement and repair, due to its excellent mechanical and crack resistance properties.

## 1. Introduction

Self-compacting concrete offers numerous advantages, including high fluidity, uniformity, stability, and the ability to fill template space without requiring external vibration during pouring. It can flow and fill the desired space under the action of its weight. However, in the early stages, it will produce a series of microcracks, which will lead to stress concentration when the concrete is subjected to external loads, thereby reducing the strength of the concrete [1,2,3,4]. In order to suppress the development of cracks in self-compacting concrete, researchers from various countries have studied the addition of various types of fiber to self-compacting concrete. The results show that adding fiber to self-compacting concrete can not only improve its low tensile strength, easy cracking, and poor ductility but also improve its strength, durability, and crack resistance performance. Therefore, the application of fiber-reinforced concrete in practical engineering is increasing. In 2020, the production capacity of basalt fibers represented by China reached 35,500 tons, with a production capacity of 10,900 tons. China’s basalt fiber production accounts for 50% of the world’s total production [5,6,7,8,9].

The workability and mechanical properties of concrete can be affected by fiber type, length, diameter, doping method, and dosage. Previously, research was often limited to the workability of fiber-reinforced concrete, such as the influence of fiber distribution on concrete fluidity and the influence of fiber length on concrete cohesion. Furthermore, mechanical properties are important in terms of whether materials or structures can meet the usage requirements, and thus, many experts have started to study the impact of various fibers on the basic mechanical properties of concrete [10,11,12]. M. G. Alberti and K. M. A. Hossain et al. studied the effects of different fiber volume ratios on the basic mechanical properties of polyacrylonitrile fiber self-compacting concrete and polyvinyl alcohol fiber self-compacting concrete and concluded that with increasing fiber content, the splitting tensile strength of self-compacting concrete gradually increases and the compressive strength gradually decreases [13,14]. To further explore the mechanical properties of fiber-reinforced concrete, M. M. Kamal et al. found through research on different levels of fiber content that increasing the content of steel–polypropylene hybrid fiber can not only improve the compressive strength of concrete but also its impact resistance [15]. Some scholars have also studied the workability of self-compacting concrete mixed with different types of fibers and found that using a large amount of fly ash can improve the workability of self-compacting concrete mixtures. However, when using lightweight self-compacting concrete with polypropylene fibers, the slump of the concrete decreases correspondingly with the increase in polypropylene fibers [16,17]. The biggest drawback of concrete is that it is prone to cracking. In order to solve the problem of the easy cracking of concrete, scholars from various countries have conducted research on the crack resistance performance of fiber-reinforced concrete [18]. A large number of experiments found that the addition of polyvinyl alcohol fibers, steel fibers, polypropylene fibers, basalt fibers, etc., to self-compacting concrete can produce a good crack resistance effect, and hybrid fiber concrete is more prone to fracture than single-long-fiber concrete [19,20,21,22]. However, the impact of global warming is becoming increasingly severe, and the sustainable development of cities has also become a focal issue [23,24]. In order to improve their environmental quality, environment-friendly fibers and natural fibers have gradually developed [25,26]. Basalt fiber is made from pure natural basalt ore and is processed at a high temperature via platinum–rhodium alloy wire drawing and a leakage plate, and is known as a pollution-free “green industrial material and new material” in the 21st century. It is characterized by high tensile strength, strong corrosion resistance, good chemical stability, good insulation, wave transmission, and radiation resistance [27,28]. Therefore, many scholars have conducted research on the relevant characteristics of a new type of composite material–basalt fiber self-compacting concrete. Through extensive experiments, it was shown that adding basalt fibers to self-compacting concrete can not only significantly improve the impact strength, toughness, and crack resistance of concrete but also improve the environmental quality [29,30,31]. Some scientists also carried out comparative experiments using two or more fibers. A. Rashno et al. studied the effect of chopped basalt fibers and polyvinyl alcohol fibers on the properties of self-compacting concrete and it was found that the flexural strength and bond strength gradually increased with the increase in fiber content [32]. Z. Celik et al. studied the performance of ordinary and self-compacting steel fiber-reinforced concrete support beams repaired using basalt fiber mesh and basalt fiber fabric. The experimental results showed that the rehabilitation of epoxy resin injection repair cracks is more effective for medium-strength support beams than high-strength support beams, and the use of basalt fiber fabric is better [33]. In addition, many experts have studied the brittle fracture performance of basalt fiber materials and found that adding appropriate basalt fiber content can reduce the shear strain, thereby reducing the degree of failure in the shear failure zone, and to some extent, delaying the cracking time of specimens [34,35,36]. The above research results further confirm that basalt fiber is an environmentally friendly material with reliable mechanical properties. Adding suitable basalt fibers into self-compacting concrete can not only improve the basic mechanical properties of concrete but also effectively prevent the formation of concrete cracks [37,38,39,40,41,42,43].

In summary, basalt fiber not only has the performance of other fibers, such as steel fiber and polypropylene fiber, but is also a new type of green building material. Therefore, it is necessary to conduct thorough research on the mechanical properties and crack resistance performance of basalt fiber self-compacting high-strength concrete. It is worth noting that the research on the crack resistance performance of basalt fiber self-compacting high-strength concrete is not yet mature, and the idea of combining innovation was used to comprehensively apply mathematical combination theory to orthogonal design experiments under multiple factors and levels, as well as the efficacy coefficient method to determine the optimal mix ratio in experimental research, which could improve the scientificity and rationality of experimental research. Therefore, the results of this research can provide reference values for future research on the application of basalt fiber self-compacting high-strength concrete in practical concrete structures, such as pavement, bridge decks, and building structure reinforcement and repair.

## 2. Material Selection and Scheme Design

### 2.1. Selection of Raw Materials

#### 2.1.1. Cement

**Figure 1:**
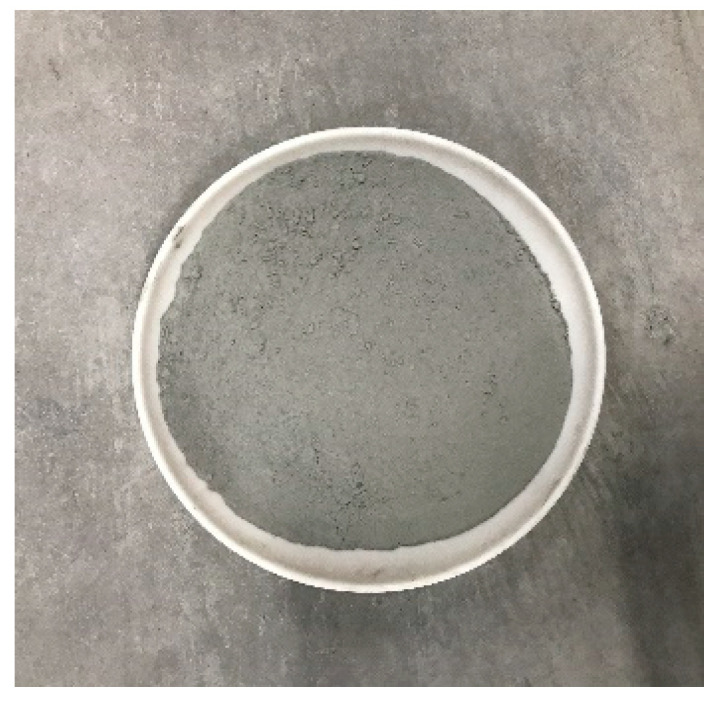
Cement.

When using cement, the main consideration is to ensure the early fluidity of freshly mixed mortar and the long-term stability of the later hardening of the slurry. This experiment used P•O 42.5 R ordinary Portland cement (produced by Harbin Yatai Cement Co., Ltd., Harbin, China), as shown in Figure 1, and its various performance indicators are shown in Table 1.

#### 2.1.2. Coarse Aggregate

**Figure 2:**
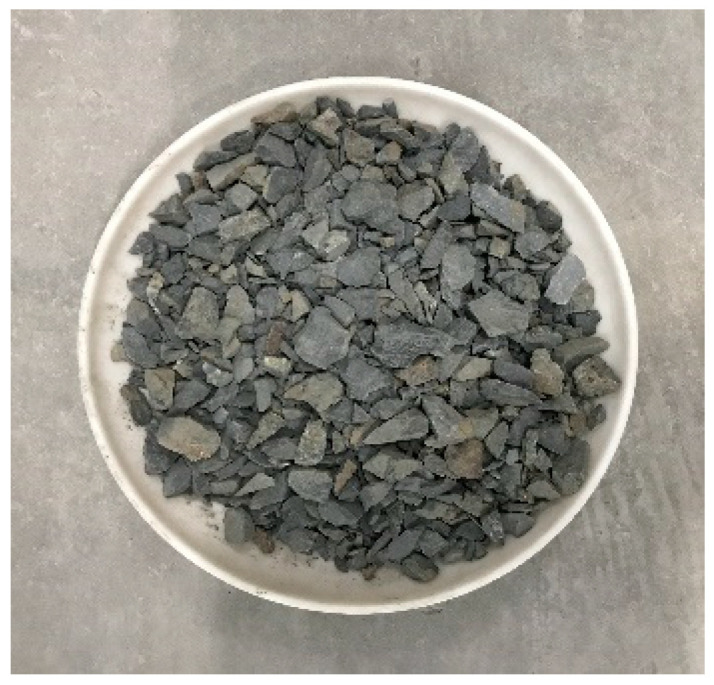
Gravel.

Coarse aggregate has a significant impact on the physical and mechanical properties and durability of self-compacting concrete. When preparing self-compacting concrete, it is advisable to use hard and rough-surfaced crushed stone as the coarse aggregate with a maximum particle size of less than 25 mm to ensure good bonding strength between the aggregate and the cement slurry. The experiment used crushed stones with a continuous grading of 5–20 mm, as shown in Figure 2. The physical properties are shown in Table 2, and the particle size distribution is shown in Table 3.

#### 2.1.3. Fine Aggregate

**Figure 3:**
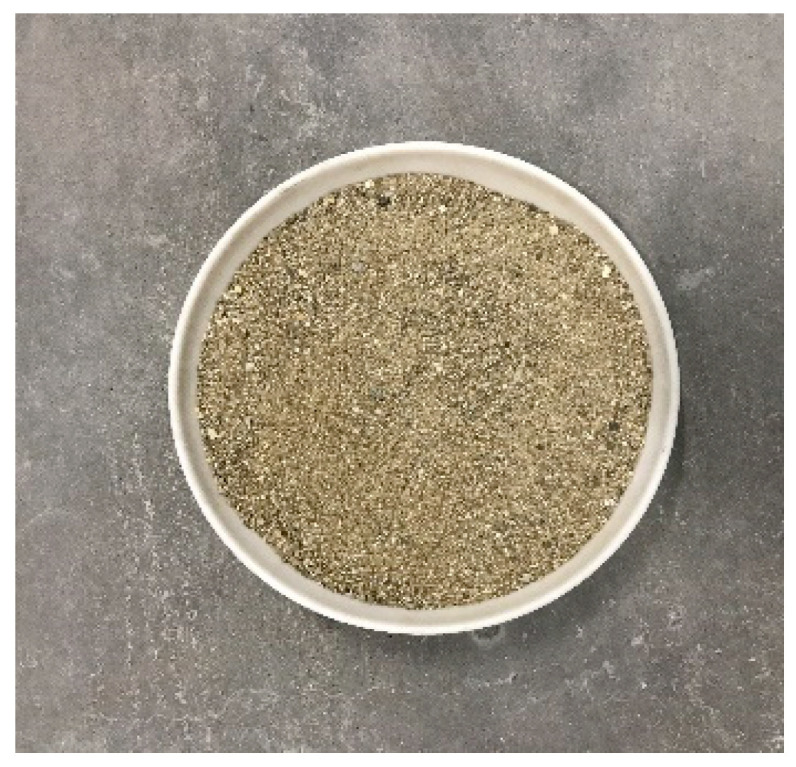
Sand.

The sand content has a significant impact on self-compacting concrete. If coarse sand is used, it is not conducive to the cohesion of the mixture; if fine sand is selected, it will increase the water required for the mixture, thereby reducing the workability of the concrete. Therefore, sand is usually chosen, where the sand grade is divided into grades Ⅰ, Ⅱ, and Ⅲ grade; choosing grade Ⅱ sand can not only ensure the fluidity of the mix but also reduce the water demand of the concrete mix. This test used the grade Ⅱ sand, as shown in Figure 3; its physical properties are shown in Table 4 and the particle gradation is shown in Table 5.

#### 2.1.4. Mineral Admixture

**Figure 4:**
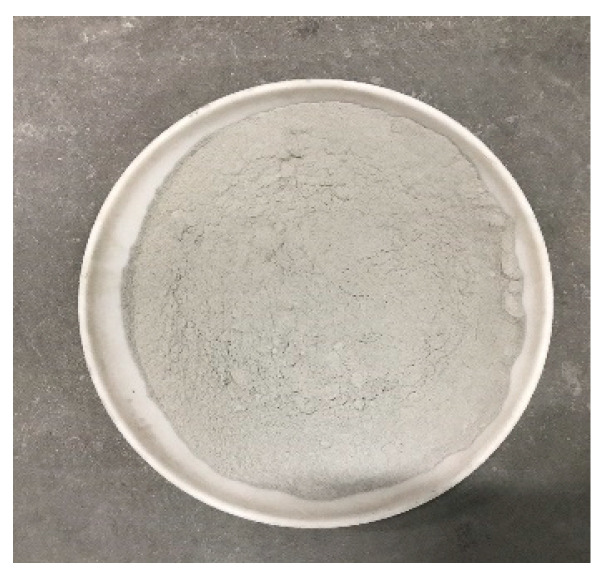
Fly ash.

For high-strength self-compacting concrete, the incorporation of a certain amount of mineral admixture is beneficial to improve the compactness and workability of concrete, while the combination of mineral admixture and cement can enhance the late strength and durability of concrete. This experiment used class I fly ash with a density of 2200 kg/m^3^ (produced at the Harbin Third Power Plant), as shown in Figure 4. Its various performance indicators are shown in Table 6.

#### 2.1.5. Admixture

**Figure 5:**
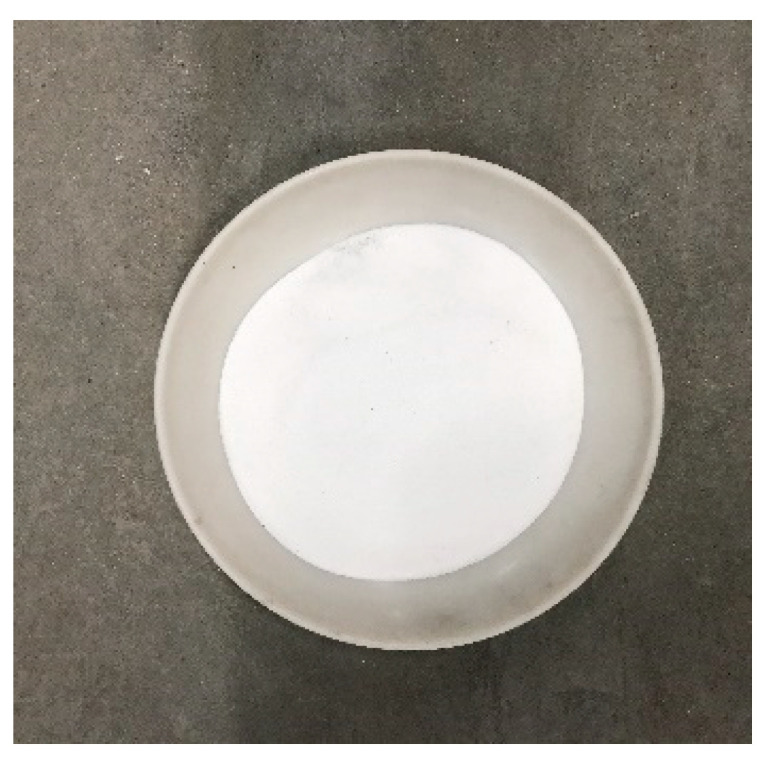
Polycarboxylic acid superplasticizer.

To achieve high flowability, gap permeability, filling ability, and high stability of self-compacting concrete, admixtures are added. The selection requirements of additives are as follows: good compatibility with cement, high water-reducing ability, and a retarding effect. This experiment used a polycarboxylic acid high-performance water-reducing agent (produced by the Sika Company, Baar, Switzerland), as shown in Figure 5, and its performance indicators are shown in Table 7.

#### 2.1.6. Fiber and Mixed Water

**Figure 6:**
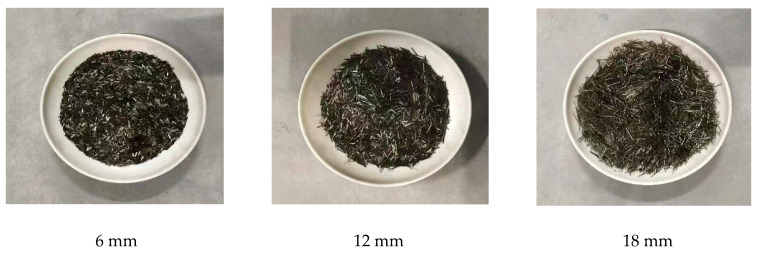
Chopped basalt fiber.

This experiment used three different lengths of short-cut basalt fibers, as shown in Figure 6 (produced by the Jiangsu Tianlong Basalt Fiber Co., Ltd., Nanjing, China), and their characteristic indicators are shown in Table 8. The mixing water should be ordinary tap water.

### 2.2. Design of Experimental Plan

#### 2.2.1. Mixing Ratio Design

This study adopted the absolute volume method for the mix design of self-compacting high-strength concrete with a level 1 performance. According to the relevant provisions of the “Technical specification for the application of self compacting concrete” (JGJ/T283-2012) [44], the specific benchmark mix design process is as follows:

(1) The weight of coarse aggregate per unit volume *M_g_*:

(1) $$M_{g}=V_{g}\rho_{g}$$

*ρ_g_—apparent density of the coarse aggregate (kg/m^3^).*

The absolute volume *V_g_* of the coarse aggregate per unit volume is determined according to the self-compacting performance class of the concrete in accordance with Table 9. Therefore, the value of *V_g_* = 0.30 m^3^ was used.

(2) Mortar volume *V_m_*:

(2) $$V_{m}=1-V_{g}$$

(3) The sand volume *V_s_* and mass *M_s_* per unit volume:

(3) $$V_{s}=V_{m}\phi_{s}$$

(4) $$M_{s}=V_{s}\rho_{s}$$

*ϕ_s_—the volume fraction of sand, taken as 0.42~0.45;*

*ρ_s_—apparent density of sand (kg/m^3^).*

(4) Slurry volume *V_p_*_:_

(5) $$V_{p}=V_{m}-V_{s}$$

(5) Apparent density of cementitious materials *ρ_b_*:

(6) $$\rho_{b}=\frac{1}{\frac{\beta }{\rho_{m}}+\frac{1-\beta }{\rho_{c}}}$$

*ρ_m_—* *apparent density of admixtures (kg/m^3^);*

*ρ_c_—* *apparent density of cement (kg/m^3^);*

*β—unit volume of concrete admixture as a percentage of the mass of the cementitious material (%)*.

(6) Preparation strength of self-compacting high-strength concrete:

(7) $$f_{cu,0}=f_{cu,k}+1.645\delta$$

*f_cu,k_—the standard value of concrete cube compressive strength, where our test took the design strength of 50 MPa;*

*δ—standard deviation (6.0 was taken as the standard deviation).*

(7) Water/binder ratio:

(8) $$\frac{m_{w}}{m_{b}}=\frac{0.42f_{ce}(1-\beta +\beta \gamma )}{f_{cu,0}+1.2}$$

*m_b_—weight of cementitious material in a unit volume of concrete (kg);*

*m_w_—water weight in a unit volume of concrete (kg);*

*β—unit volume of concrete admixture as a percentage of the mass of the cementitious material (%);*

*f_ce_—the 28 d measured compressive strength of cement (MPa), which can be taken as 1.1 times the cement strength value when it is unknown;*

*γ—the cementation coefficient of mineral admixture; fly ash (β ≤ 0.3) can be taken as 0.4.*

(8) Mass per unit volume of cementitious material *m_b_*:

(9) mb=Vp−Va1ρb+mwmbρw

*V_a_—the volume of air introduced into a unit volume of concrete (L), we took V_a_ to be 15 L;*

*ρ_w_—we used 1000 kg/m^3^.*

(9) The mass of mineral admixture per unit volume *m_m_*, the mass of cement m_c_, and the mass of water *m_w_*:

(10) $$m_{m}=\beta m_{b}$$

(11) $$m_{c}=m_{b}-m_{m}$$

(12) mw=mb×mwmb

(10) The dosage of additives can be calculated using the following formula:

(13) $$m_{ca}=\alpha m_{b}$$

*m_ca_—mass of the admixture in a unit volume of concrete (kg);*

*α—admixture per unit volume of concrete as a percentage of the total cementitious material (%).*

Through theoretical calculations and repeated trial formulations, the C50 self-compacting high-strength concrete ratio was obtained, and the specific parameters are shown in Table 10.

#### 2.2.2. Orthogonal Experimental Design

Orthogonal experimentation is a design method that uses mathematical combination theory to study the situation of multiple factors and levels. This method involves selecting representative test points from comprehensive tests through orthogonality. There are many factors that affect the strength of concrete, such as cement strength, aggregate particle size, admixtures, mineral admixtures, and curing temperature. This study considered the water binder ratio (0.30, 0.32, 0.34), fiber volume fraction (0.1%, 0.2%, 0.3%), fiber length (6 mm, 12 mm, 18 mm), and fly ash content (20%, 25%, 30%) as the main influencing factors, denoted as A, B, C, and D, respectively. Three levels of factors were taken for each experimental factor, and the factor level table is shown in Table 11. Without considering the interaction between the factors, the L9 (3^4^) orthogonal table was selected for the orthogonal test based on the factor levels, as shown in Table 12.

#### 2.2.3. Preparation and Maintenance of Specimens

**Figure 7:**
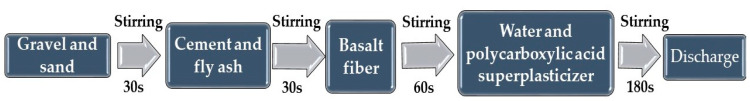
Test piece production process.

We conducted tests in accordance with relevant concrete specifications [45,46], including the compressive strength test, splitting tensile strength test, and flexural strength test. The working performance requirements of self-compacting concrete are high. When fibers are added to the concrete, the uniform distribution of fibers has a significant impact on the performance of self-compacting concrete. Therefore, the feeding sequence and mixing process are very important for preparing basalt fiber self-compacting high-strength concrete. This experiment adopted the dry mixing method for mixing. In order to prevent voids in the mixture and ensure full contact between each mixture, the mixture was gradually mixed in the order of input and the particle size was gradually decreased from large to small. The specific process is shown in Figure 7, and all the operation processes were completed within 5 min.

After the fiber concrete was mixed, it was loaded into the mold, the surface was smoothed, and the concrete was placed in a 20 ± 5 °C environment. After 24 h of curing, the mold was removed and the test blocks were numbered. Then, the test blocks were placed in a curing box, controlled at a temperature of 20 ± 3 °C, and the relative humidity was controlled to be not less than 95%. After 28 days of curing, the test pieces were taken out and tested sequentially. The size and number of test pieces required for each test are shown in Table 13.

## 3. Study of the Mechanical Properties of Basalt Fiber Self-Compacting High-Strength Concrete

### 3.1. Compressive Strength Test

#### 3.1.1. Test Method

**Figure 8:**
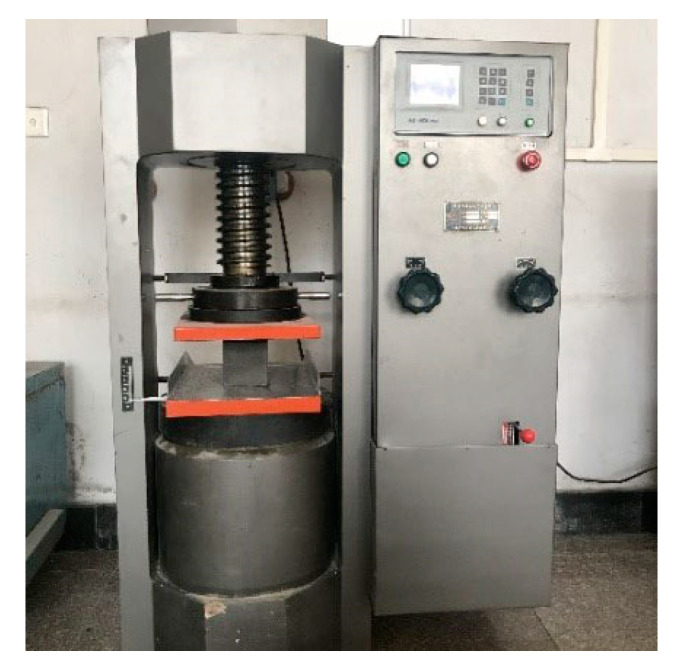
Loading chart for the compressive strength test.

The compressive strength test of the basalt fiber self-compacting high-strength concrete adopted a screen display hydraulic pressure testing machine, where the testing equipment is shown in Figure 8. 

**Figure 9:**
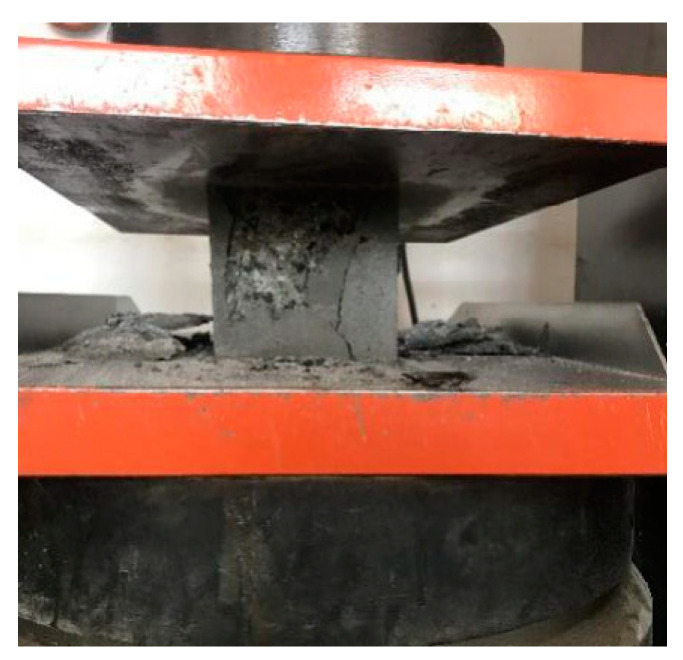
Failure of a compressive strength specimen.

The loading steps for this experiment were as follows:

(1) We took out the specimen whose maintenance age reached 28 d and wiped the surface dry.

(2) The pressure-bearing surface of the test piece was placed perpendicular to the top surface during molding on the lower pressing plate. The center position of the test piece was aligned with the center of the lower pressing plate. We started the testing machine, and when the upper pressing plate was close to the test piece, we pressed the ball seat to ensure that it evenly touched the test piece.

(3) In this study, the strength grade of the concrete was C50, the test loading was continuous, and the loading rate used was 0.5~0.8 MPa per second.

(4) When the specimen was deformed, the accelerator of the testing machine was controlled until the specimen is damaged, and then the load should be recorded. The compressive failure morphology of the specimen is shown in Figure 9.

#### 3.1.2. Test Results and Analysis

(1) Calculation of the compressive strength of concrete:

(14) $$f_{cu}=\frac{F}{A}$$

*ƒ_cu_—compressive strength value (MPa);*

*F—destructive load value (N);*

*A—pressure-bearing surface area (mm^2^).*

(2) The compressive strength value met the following requirements:

① The result was accurate to 0.1 MPa.

② The strength value of this group was the average value of three specimens.

③ If the maximum or minimum value of the test block was compared with the middle value and one of the extremum values varied from the middle value by 15%, the middle value was taken as the compressive strength value; if both the extremum values varied by more than 15% from the middle value, the group results were invalid.

④ This test selected the dimension side length of a 100 mm non-standard cube specimen, and thus, the results were multiplied by the size conversion factor of 0.95.

The results of the experimental data are listed in Table 14, and the results of the extreme difference analysis are shown in Table 15.

**Figure 10:**
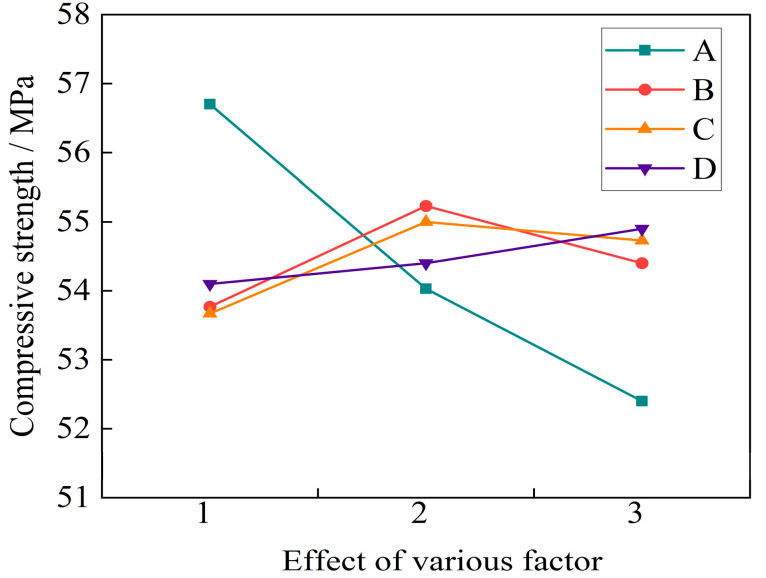
Effect of various factor levels on the 28 d compressive strength.

From Figure 10, it can be seen that when the water binder ratio was 0.3, the maximum compressive strength of the basalt fiber high-strength self-compacting concrete at 28 days was 56.7 MPa. When the water binder ratio reached 0.34, the compressive strength at 28 days decreased by 7.5%, that is, as the water binder ratio increased, the compressive strength at 28 days gradually decreased.

As the fiber volume fraction increased, the compressive strength first increased and then decreased. When the volume fraction was 0.2%, the maximum strength reached 55.23 MPa, and a turning point occurred. When the fiber volume fraction was 0.3%, the strength decreased by 1.5%, indicating that the optimal fiber volume fraction affected the 28 d compressive strength of the fiber self-compacting high-strength concrete. The fiber length also had an impact on the compressive strength of concrete, when the fiber length increased from 6 mm to 12 mm, the 28 d compressive strength showed a slow growth trend, but when the fiber length was 18 mm, the compressive strength slightly decreased, indicating that adding too long fibers had an adverse effect on the compressive strength of concrete.

The influence of fly ash content on the compressive strength was relatively small, and the 28 d compressive strength of basalt fiber self-compacting high-strength concrete slowly increased with the addition of the fly ash content.

From Table 15, it can be seen that the main and secondary order of factors affecting the 28 d compressive strength of the basalt fiber self-compacting high-strength concrete was as follows: water binder ratio > fiber volume ratio > fiber length > fly ash content. The most significant weights of the factors on compressive strength were a water binder ratio of 0.3, fiber volume ratio of 0.2%, fiber length of 12 mm, and fly ash content of 30%. When the assessment index was the 28 d compressive strength, the optimal mix ratio of the basalt fiber self-compacting high-strength concrete was A_1_B_2_C_2_D_3_.

### 3.2. Splitting Tensile Strength Test

#### 3.2.1. Test Method

**Figure 11:**
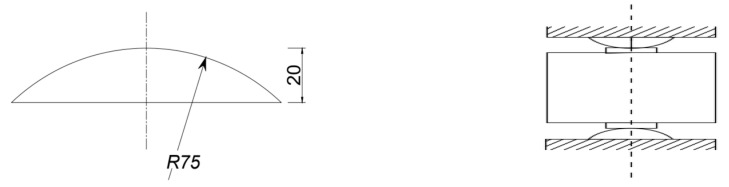
Schematic of the split tensile test device.

The splitting tensile strength testing equipment for basalt fiber self-compacting high-strength concrete was the same as the compressive strength, but it was necessary to place cushion blocks and strips on and below the pressure-bearing plate of the press. The cushion blocks were made of steel material with a radius of 75 mm, the width of the cushion strips was 20 mm, and the thickness was 3–4 mm. The lengths of the cushion blocks and strips should not be less than the side length of the test piece, and the cross-sectional dimensions are shown in Figure 11.

**Figure 12:**
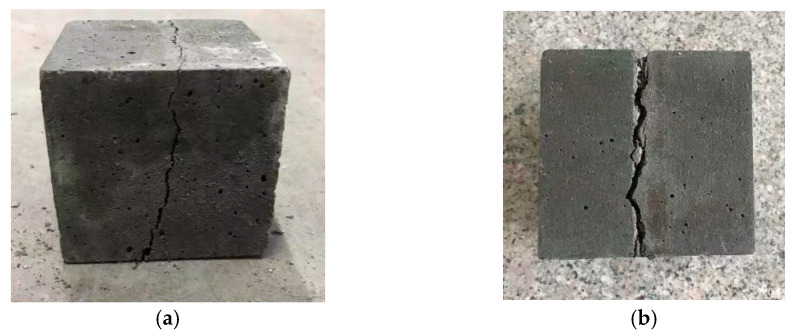
Fracture of a split tensile strength specimen. (**a**) lateral; (**b**) bottom.

The specific test steps were as follows:

We took out the specimens with a curing age of 28 days and wiped their surfaces clean. Then, we placed the test piece in the center of the lower pressing plate of the testing machine and aligned the cushion block and strip with the centerline of the upper and lower surfaces of the test piece. Then, the testing machine was started, and we maintained continuity in the loading process, where it was controlled between 0.05 and 0.08 MPa/s. When the specimen was about to break, we adjusted the throttle until the specimen broke and recorded the load value at this time. The splitting tensile failure morphology of the specimen is shown in Figure 12.

#### 3.2.2. Test Results and Analysis

(1) The formula for the concrete splitting tensile strength is as follows:

(15) $$f_{ts}=\frac{2F}{\pi A}=0.637\frac{F}{A}$$

*f_ts_—splitting tensile strength value (MPa);*

*F—failure load value (N);*

*A—splitting surface area (mm^2^).*

(2) The splitting tensile strength value met the following requirements:

① The result was accurate to 0.01 MPa;

② The value requirements were the same as the compressive strength;

③ This experiment selected non-standard cubic specimens with a side length of 100 mm, and the results were multiplied by a size conversion factor of 0.85.

The experimental data results are listed in Table 16, and the range analysis results are shown in Table 17.

**Figure 13:**
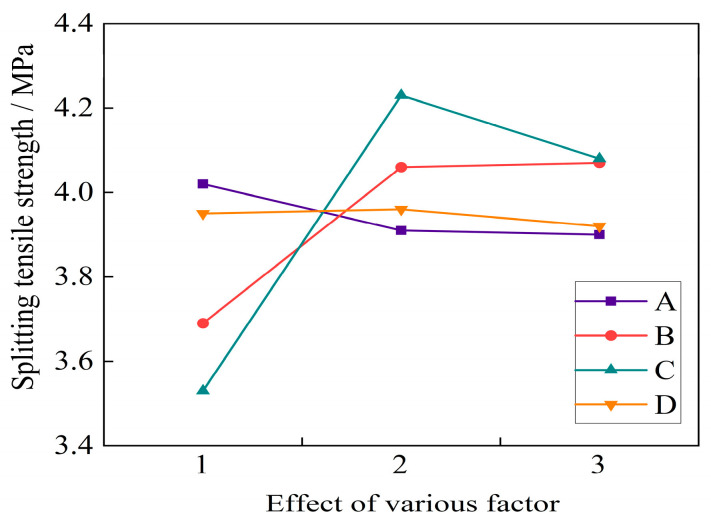
Effect of various factor levels on the 28 d splitting tensile strength.

From the test results in Figure 13, it can be seen that the effect on 28 d splitting tensile strength showed a slow downward trend when the water binder ratio increased from 0.3 to 0.34. When the water binder ratio was 0.3, the splitting compressive strength of the basalt fiber self-compacting high-strength concrete is the highest, that is, the optimal factor level of water binder ratio was 0.3. When the volume fraction of basalt fibers was increased from 0.1% to 0.2%, the increase in strength was relatively large. When the fly ash was added at 0.3%, the strength change in the self-compacting concrete showed a gentle increase. As the fiber length increased, the splitting tensile strength of the self-compacting high-strength concrete showed a trend of first increasing and then decreasing. When the fiber length increased from 6 mm to 12 mm, the splitting tensile strength first increased by 19.8% and then decreased by 3.5%.

From Table 3, Table 4, Table 5, Table 6 and Table 7, it can be seen that the fiber length was the main factor that affected the splitting tensile strength of the fiber self-compacting high-strength concrete. The 28 d splitting tensile strength gradually decreased with the increase in fly ash content. When the fly ash content increased from 20% to 30%, the strength only decreased by 0.8%, indicating that the fly ash content had little effect on the strength change. The main and secondary order of factors that affected the 28 d splitting tensile strength of the basalt fiber self-compacting high-strength concrete was as follows: fiber length > fiber volume fraction > water binder ratio > fly ash content. The most significant influence weights of the various factors on the splitting tensile strength were the water binder ratio of 0.3, fiber volume ratio of 0.3%, fiber length of 12 mm, and fly ash content of 25%. When the assessment index was the 28 d splitting tensile strength, the optimal mix ratio of the basalt fiber self-compacting high-strength concrete was A_1_B_3_C_2_D_2_.

### 3.3. Flexural Strength Test

#### 3.3.1. Test Method

**Figure 14:**
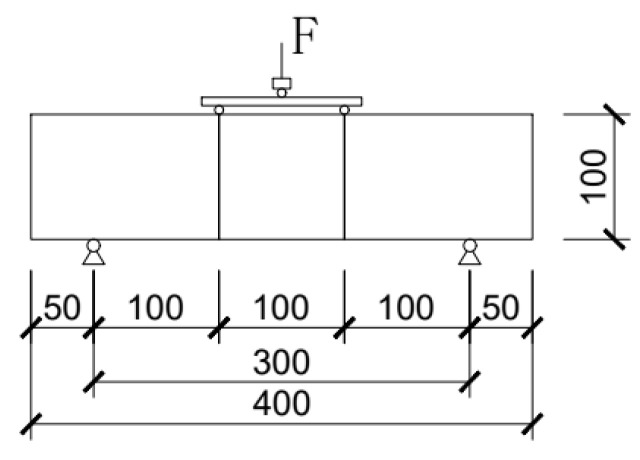
Schematic diagram of the flexural strength test device.

**Figure 15:**
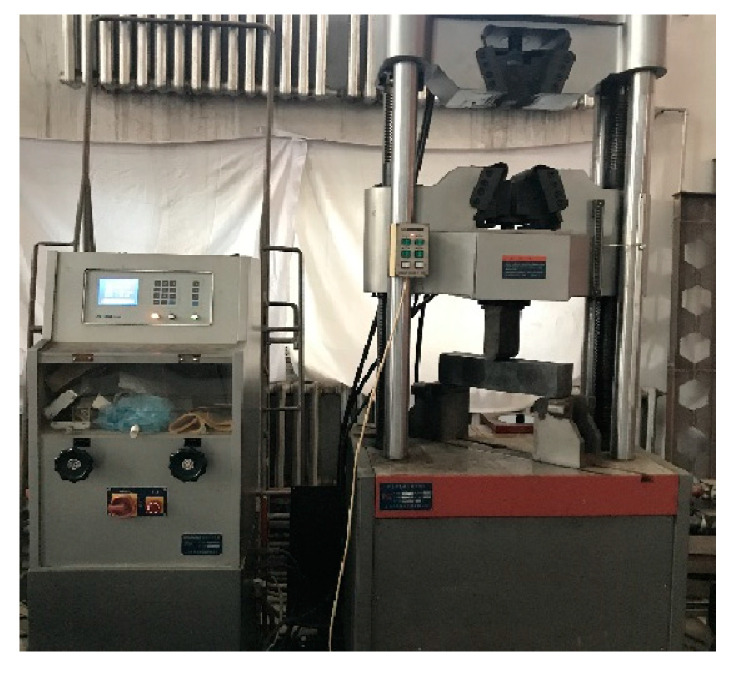
Loading diagram of the flexural strength test.

The bending strength test of the basalt fiber self-compacting high-strength concrete was conducted using a screen display hydraulic universal testing machine, where the bending test device is shown in Figure 14 and Figure 15.

The specific test steps were as follows:

We removed the specimens after a curing period of 28 days and dried their surfaces. Then, we selected the side of the specimen during molding as the testing surface, placed the specimen according to the device diagram, and installed it with a deviation of no more than 1 mm on the left and right. We ensured that the cylindrical contact surface was stable on the support and testing surface.

**Figure 16:**
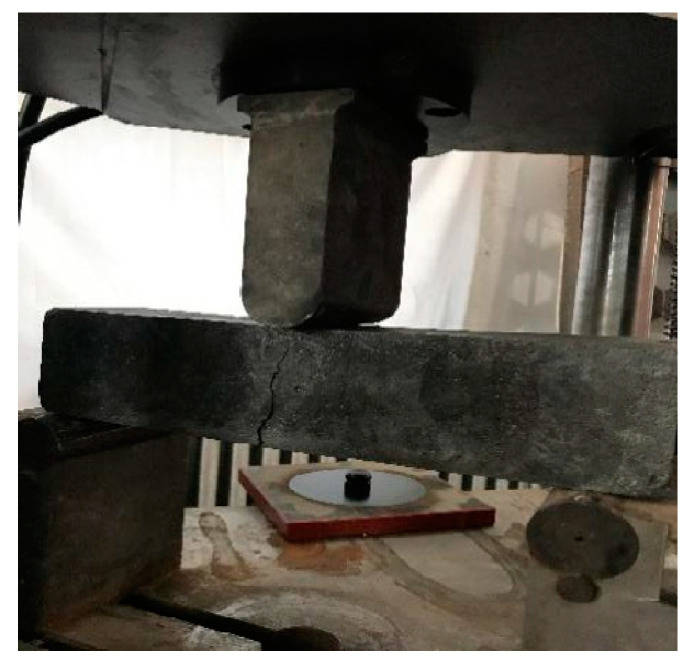
Failure of a flexural strength specimen.

When applying the load, it was necessary to accelerate uniformly and continuously, which was controlled to be between 0.05 and 0.08 MPa/s. When the specimen was damaged, the throttle was controlled until the specimen was completely damaged, and then the load value was recorded. The flexural failure morphology of the specimen is shown in Figure 16.

#### 3.3.2. Test Results and Analysis

(1) If the fracture position of the lower edge of the specimen is between two concentrated load action lines, the flexural strength is calculated as follows:

(16) $$f_{f}=\frac{FL}{bh^{2}}$$

*f_f_—flexural strength value (MPa);*

*F—failure load value (N);*

*L—span between supports (mm);*

*h—section height (mm);*

*b—section width (mm).*

(2) The flexural strength value met the following requirements:

① The result was accurate to 0.1 MPa.

② If the fracture position of one specimen was not between the two concentrated loads, the results of the other two specimens were used for the strength value. If the difference between these two measured values was less than 15% of the smaller value, the average value of the two specimens was taken as the flexural strength of the group. Otherwise, the results were considered invalid.

③ The selected size for this experiment was 100 mm × 100 mm × 400 mm. The result of a 400 mm non-standard specimen was multiplied by a size conversion factor of 0.85.

The experimental data results are listed in Table 18, and the range analysis results are shown in Table 19.

**Figure 17:**
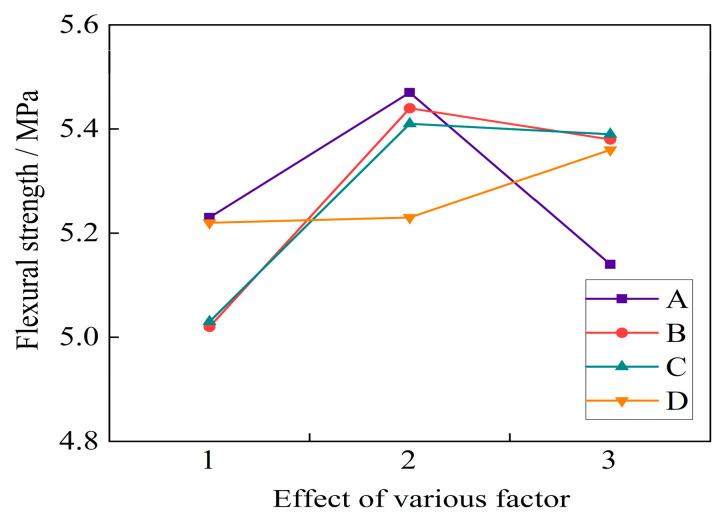
Effect of various factor levels on the 28 d flexural strength.

From Figure 17, it can be seen that the 28 d flexural strength of basalt fiber self-compacting high-strength concrete showed a trend of first increasing and then decreasing with the increase in water binder ratio. When the water binder ratio was 0.32, the maximum flexural strength reached 5.47 MPa. As the fiber volume fraction increased, the flexural strength reached its peak at a fiber volume fraction of 0.2%. If the dosage was further increased, the flexural strength began to decrease, indicating that there was an optimal fiber dosage that affected the strength of fiber self-compacting high-strength concrete. When fibers with a length of 12 mm were added, the flexural strength at 28 days reached 5.41 MPa, which was an increase of 7.6% compared with the fiber length of 6 mm; when the fiber length increased to 18 mm, the bending strength showed a slight downward trend, indicating the existence of an optimal fiber length. With the addition of fly ash, the 28-day flexural strength initially remained almost unchanged. When the dosage increased from 25% to 30%, the growth rate of flexural strength increased. When the fly ash dosage was 30%, the maximum flexural strength was 5.39 MPa.

According to Table 19, the main and secondary order of factors that affected the 28 d flexural strength of basalt fiber self-compacting high-strength concrete was as follows: fiber volume ratio > fiber length > water binder ratio > fly ash content. The most significant influence weights of various factors on flexural strength were the water binder ratio of 0.32, fiber volume ratio of 0.2%, fiber length of 12 mm, and fly ash content of 30%. When the assessment index was 28 d flexural strength, the optimal mix ratio of basalt fiber self-compacting high-strength concrete was A_2_B_2_C_2_D_3_.

## 4. Study of the Crack Resistance of the Basalt Fiber Self-Compacting High Strength Concrete

### 4.1. Determination of the Optimal Mix Ratio

According to the efficacy coefficient method, the overall evaluation of the performance indicators of basalt fiber self-compacting high-strength concrete was carried out. Based on the results of the previous mechanical performance tests, the corresponding efficacy coefficients and total efficacy coefficients d of each assessment indicator were calculated to obtain the optimal mix proportion of basalt fiber self-compacting high-strength concrete with a comprehensive performance.

(17) $$d=\sqrt[{m}]{d_{1}d_{2}\cdots d_{m}}$$

The efficacy coefficient of each assessment indicator is *d_i_* (*i* = 1, 2,..., *m*), and each efficacy coefficient *d_i_* is the ratio of the indicator value to the optimal indicator value of the group’s experimental results. The total efficacy coefficient *d* is used to represent the advantages and disadvantages of m assessment indicators. The larger *d* is, the more favorable the efficacy of the group of experiments on m indicators.

From Table 20, it can be seen that the total efficacy coefficient *d* = 0.988 of test BF-2 was the highest, and the corresponding test condition was A1B2C2D2. From the magnitudes of the range R and K values in Table 21, it can be seen that the factors that affected the comprehensive performance of basalt fiber self-compacting high-strength concrete were ranked as follows: fiber length > fiber volume fraction > water binder ratio > fly ash content. Among the four factors, the fiber length had the greatest impact on the comprehensive performance of the self-compacting high-strength concrete, followed by the fiber volume fraction, followed by the water binder ratio, and the factor with the smallest impact was the amount of fly ash content. The best level of the influencing factors A, B, C, and D was A_1_B_2_C_2_D_3_. In summary, the optimal experimental plan for the comprehensive performance of the basalt fiber self-compacting high-strength concrete was as follows: a water binder ratio of 0.3, basalt fiber volume ratio of 0.2%, fiber length of 12 mm, and fly ash content of 30%.

### 4.2. Test Method

This study selected an improved plate constraint method, namely, the knife-edge-induced constraint method. Based on the testing results of the mechanical properties of the basalt fiber self-compacting high-strength concrete in the early stage, the optimal mix ratio was obtained as A1B2C2D3. Therefore, under the conditions of a water binder ratio of 0.3 and a fly ash content of 30%, the early crack resistance performance of the C50 basalt fiber self-compacting concrete with basalt fiber volume ratios of 0.1%, 0.2%, and 0.3% and basalt fiber lengths of 6 mm, 12 mm, and 18 mm was studied.

**Figure 18:**
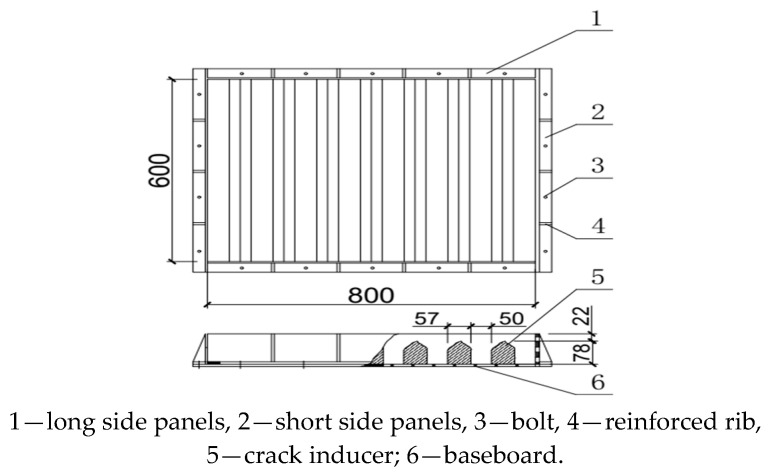
Schematic diagram of the early crack resistance test device.

The test mold used for the edge induction constraint method was an 800 mm × 600 mm × 100 mm steel mold welded with channel steel on all four sides and embedded with bolts on the bottom plate. The mold was equipped with seven crack inducers, which were parallel to the short side of the mold. The bottom plate was laid with steel plates (plate thickness > 5 mm), and a plastic film was placed on top of it as an isolation layer. The experimental setup is shown in Figure 18.

The test steps were as follows:

We applied template oil evenly on the inside and bottom of the mold, poured the mixed self-compacting concrete into the mold, leveled the concrete, and the surface was slightly higher than the mold frame. The curing conditions of the specimen were as follows: temperature 20 ± 2 °C and relative humidity 60 ± 5%.

After the specimen had formed for 30 min, we immediately adjusted a fan to blow directly onto the surface of the specimen so that the wind direction was parallel to 100 mm above the center of the specimen surface and adjusted the wind speed to 5 ± 0.5 m/s along the blade direction.

After 24 h, we identified the cracks on the specimen, measured the lengths of the cracks with a steel ruler, and took the straight distance between the two ends of the crack as the crack length. When there were two cracks on a knife edge, the lengths of the two cracks were added together to convert them into one crack.

The maximum width of each crack was measured using a reading microscope that was 100 times the width of the crack.

When measuring the development of cracks after 24 h, the main tests were the number of cracks, maximum crack width, and total crack area. Based on the above test results and crack reduction coefficient as evaluation indicators, we explored the crack resistance effect of the fiber-reinforced self-compacting concrete.

### 4.3. Test Results and Analysis

(1) Average cracking area per crack:

(18) $$a=\frac{1}{2N}\sum_{i=1}^{n}w_{i}l_{i}$$

(2) Number of cracks per unit area:

(19) $$b=\frac{N}{A}$$

(3) Total cracking area per unit area:

(20) c=ab

(4) Crack reduction coefficient:

(21) $$\eta =\frac{A_{mcr}-A_{fcr}}{A_{mcr}}$$

*ω_i_—maximum width of the i-th crack (0.01 mm);*

*l_i_—length of the i-th crack (0.01 mm);*

*N—total number of cracks (pieces);*

*A—plate area (mm^2^);*

*a—average cracking area of each crack;*

*b—number of cracks per unit area (0.1/m^2^);*

*c—total cracking area per unit area (mm^2^/m^2^);*

*A_fcr_—nominal total area of cracks in specimens containing fibers (mm^2^);*

*A_mcr_—nominal total area of cracks in specimens without fibers (mm^2^).*

**Figure 19:**
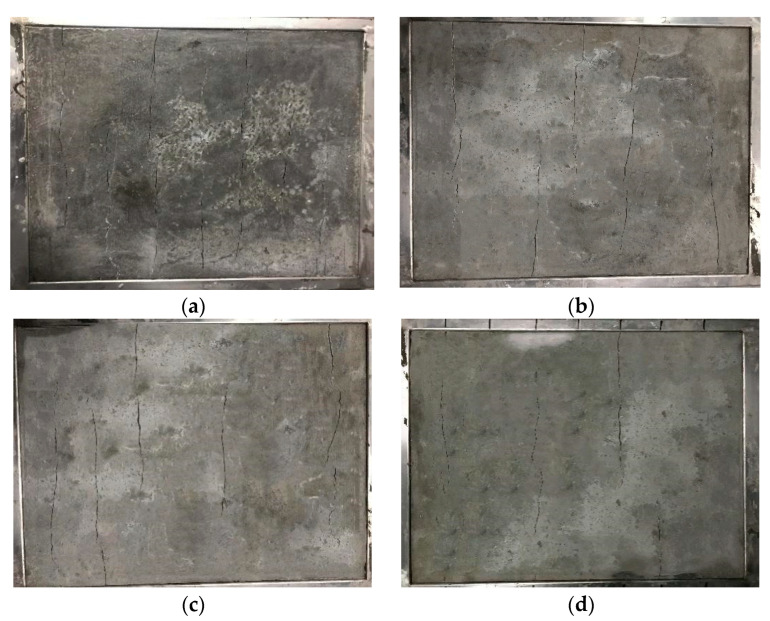

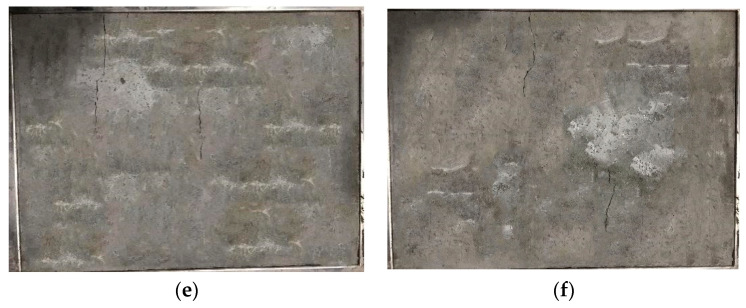
Morphologies of the cracks in the concrete specimens of each group. (**a**) NC; (**b**) K-0.1–12; (**c**) K-0.2–6; (**d**) K-0.2–12; (**e**) K-0.2–18; (**f**) K-0.3–12.

The experimental data results calculated based on the above formula are listed in Table 22 and Table 23. The crack morphology of each group of concrete specimens is shown in Figure 19a–f.

#### 4.3.1. Effect of the Fiber Volume Fraction on the Cracks in Self-Compacting High-Strength Concrete

**Figure 20:**
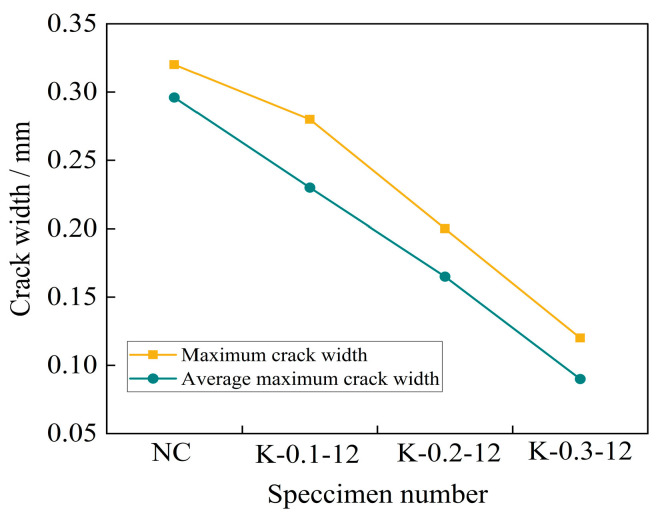
Curves of the crack width and fiber volume ratio.

From Figure 20, it can be seen that under a certain fiber length, the maximum crack width of each group of concrete specimens and the average crack width of each blade of the specimens showed a decreasing trend with the increase in fiber volume fraction. The maximum crack width of the self-compacting concrete specimen without fiber addition was 0.32 mm. When the fiber volume fraction increased from 0.1% to 0.3%, the maximum crack width of each specimen decreased by 12.5%, 37.5%, and 62.5% compared with the reference concrete specimen. The average maximum crack width of each cutting edge was 0.3 mm, and the average maximum crack width of each cutting edge of the specimen decreased by 22.3%, 44.3%, and 69.6% compared with the reference concrete specimen. It can be seen that the addition of fibers effectively suppressed the spread of the crack width.

**Figure 21:**
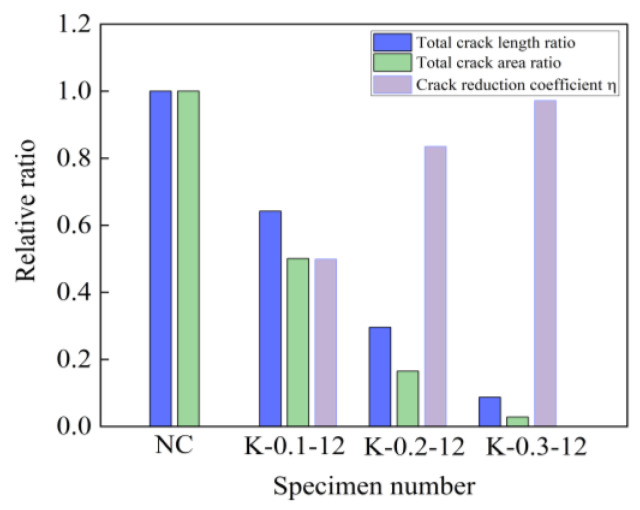
Total crack length ratio, total crack area ratio, and crack reduction coefficient *η*.

According to the variation pattern of the total crack length in Figure 21, it can be seen that the total crack length of the self-compacting concrete specimens mixed with fibers was smaller than that of the reference concrete, and when the fiber volume ratio increased, the total crack length gradually decreased. The total cracking area of cracks was a comprehensive indicator for evaluating the early cracking performance of specimens. As the fiber volume fraction increased, the total cracking areas of cracks significantly decreased to 49.9%, 83.5%, and 97.2% lower than the benchmark concrete specimen. For the crack reduction coefficient *η*, with the increase in fiber volume fraction, there was a continuous growth trend, and when the fiber volume fraction was 0.3%, *η* ≈ 1. Early cracks in concrete specimens were almost invisible. From the above analysis of results, it can be concluded that the addition of basalt fibers could improve the generation of cracks in cement-based materials. This was due to the fiber adsorbing a large amount of cement slurry. This can improve the gripping force between fibers and concrete, thereby enhancing the interfacial adhesion of the fiber matrix, leading to the fibers being able to fully suppress concrete cracking.

#### 4.3.2. Effect of Fiber Length on Cracks in the Self-Compacting High-Strength Concrete

**Figure 22:**
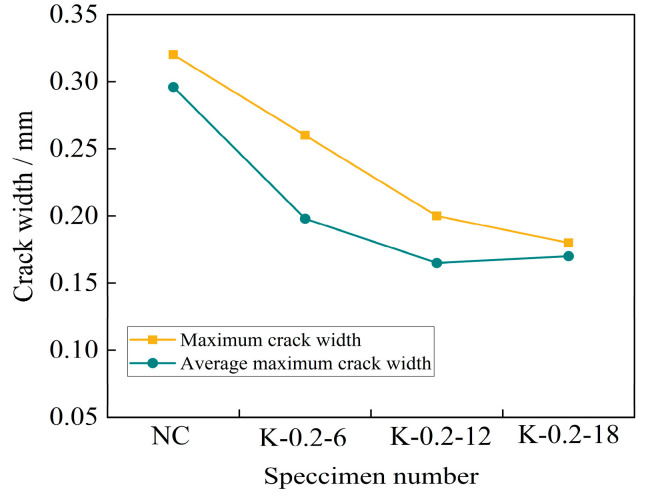
Curves of crack width and fiber length of the specimens.

Figure 22 shows that when the fiber volume fraction was fixed at 0.2%, basalt fibers of different lengths effectively prevented the maximum crack width of concrete from penetrating, and the maximum crack width of concrete gradually decreased with the increase in fiber length. Compared with the benchmark concrete, the fiber-reinforced concrete specimens with 6 mm, 12 mm, and 18 mm fibers decreased by 18.8%, 37.5%, and 43.8%, respectively. As the fiber length increased, the average maximum crack width of the concrete specimens showed a turning point at a fiber length of 12 mm; when 18 mm fibers were added, the crack width slightly increased. The reason for this phenomenon was that during the mixing process of self-compacting concrete, agglomeration occurs due to uneven mixing due to long fibers, which resulted in an increase in the average maximum crack of the concrete specimen. Therefore, excessive fiber length was not conducive to the early crack resistance of the self-compacting concrete.

**Figure 23:**
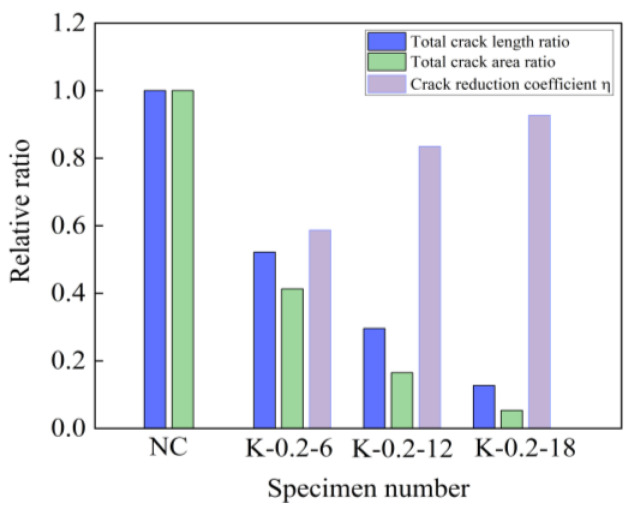
Total crack length ratio, total crack area ratio, and crack reduction coefficient *η*.

As shown in Figure 23, fiber length was also the main influencing factor determining the total length and area of cracks. When the fiber length was 6 mm, the total cracking area of the self-compacting concrete was 454.96 mm^2^, which was 58.7% lower than the benchmark concrete and the crack reduction coefficient *η* reached approximately 0.6; the self-compacting high-strength concrete specimens with fiber lengths of 12 mm and 18 mm were added, and the total cracking area decreased by 83.5% and 92.7%, respectively, with a crack reduction coefficient *η* ≈ 1. Overall, the addition of basalt fibers of different lengths significantly improved the crack resistance performance of self-compacting high-strength concrete. From the experimental phenomenon, it can be observed that excessive fiber length also had a certain adverse effect on concrete cracking. Therefore, there was an optimal range of fiber length effects on the cracking performance of the self-compacting concrete.

## 5. Conclusions

Through experimental research on the basic mechanical properties and crack resistance performance of basalt fiber self-compacting high-strength concrete, the main conclusions were as follows:

(1) The fiber volume fraction was the main factor that affected the compressive and flexural strengths of the self-compacting high-strength concrete. When an appropriate amount of basalt fiber material was added to the self-compacting high-strength concrete, it significantly improve the compressive and flexural strength of the concrete. However, when the fiber content exceeded or is less than the appropriate amount, it reduced the compressive and flexural strength of the concrete.

(2) The fiber length was the main factor that affected the splitting tensile strength of the self-compacting high-strength concrete. When a suitable length of basalt fiber material was added to the self-compacting high-strength concrete, the splitting tensile strength of the concrete was significantly improved. However, when the fiber length exceeded or was less than the appropriate length, the splitting tensile strength of the concrete was reduced.

(3) There was an optimal value or range for the influence of fiber length and fiber volume ratio on the performance of concrete.

(4) Adding appropriate fiber length and fiber volume fraction could effectively suppress the spread and penetration of crack width, thereby improving the plasticity and toughness of the self-compacting high-strength concrete.

The above conclusions are basically consistent with the results found in the literature [5,7,22,35], demonstrating the accuracy of the experimental research results. However, due to limited experimental conditions, in the study of the crack resistance of the basalt fiber self-compacting high-strength concrete, crack observation was measured using a normal 100-times reading microscope. However, existing scientific crack research can binarize the taken crack photos through image processing to extract effective crack data. In summary, adding appropriate basalt fiber materials to concrete can not only improve the basic mechanical properties of concrete but also significantly improve its crack resistance. This will be beneficial for increasing the service life of concrete structures and is important for promoting national economic construction.

## Figures and Tables

**Table 1:** Cement performance indicators.

Testing Items	Fineness (%)	Loss on Ignition (%)	MgO (%)	SO_3_ (%)	Flexural Strength (MPa)		Compressive Strength (MPa)	
					3 d	28 d	3 d	28 d
Standard value	≤10.0	≤5.0	≤5.0	≤3.5	≥3.5	≥6.5	≥17.0	≥42.5
Actual measured value	2.0	4.07	1.47	1.75	5.1	8.3	24.4	50.1

**Table 2:** Physical properties of gravel.

Mud Content (%)	Apparent Density (kg/m^3)^	Bulk Density (kg/m^3^)	Needle and Flake Content (%)	Crushing Value (%)	Porosity (%)
1.2	2700	1620	2.8	9.6	44

**Table 3:** Particle grading.

Gradation	Nominal Diameter (mm)	Sieve Diameter (mm)	Sieve Residue (%)	Accumulated Sieve Residue (%)
Gap gradation	5~20	16.0	35.7	35.7
		9.50	18.6	54.3
		4.75	38.9	93.2
		2.36	6.8	100

**Table 4:** Physical properties of sand.

Mud Content (%)	Fineness Modulus	Apparent Density (kg/m^3^)	Bulk Density (kg/m^3^)	Porosity (%)
1.4	2.6	2600	1450	40

**Table 5:** Particle grading.

Sieve Diameter (mm)	5.0	2.5	1.25	0.630	0.315	0.16	<0.16
Sieve residue (%)	0	7.35	13.33	31.67	31.83	14.73	1.09
Accumulated sieve residue (%)	0	7.35	20.68	52.35	84.18	98.91	100

**Table 6:** Fly ash performance indicators.

Testing Items	(45 μm Square Hole Sieve Residue Value) Fineness (%)	Water Demand Ratio (%)	Loss on Ignition (%)	Moisture Content (%)	SO_3_ (%)
Standard value	≦12.0	≦95	≦5.0	≦1.0	≦3.0
Actual measured value	8.7	91	2.1	0.3	0.5

**Table 7:** Polycarboxylic acid superplasticizer index.

Model	Moisture Content	NaSO_4_	Cl^−^	Water Reduction Rate
Viscocrete 530 p	3%	0.6%	0.01%	28%

**Table 8:** Physical and mechanical properties of basalt fiber.

Monofilament Diameter (μm)	Length (mm)	Density (kg/m^3^)	Tensile Strength (MPa)	Elastic Modulus (GPa)	Elongation (%)
16	6, 12, 18	2600	3000	81.9	3.22

**Table 9:** Volume of the coarse aggregate in concrete per unit volume.

Performance Level	Ⅰ	Ⅱ	Ⅲ
Volume of coarse aggregate (m^3^)	0.28~0.30	0.30~0.33	0.32~0.35

**Table 10:** Proportion of self-compacting high-strength concrete (kg/m^3^).

Cement	Sand	Cobble	Fly Ash	Water
422	815.5	825.5	141	180

**Table 11:** Factors and levels.

Horizontal Factor	Water Binder Ratio (W/B): A	Fiber Volume Fraction: B (%)	Fiber Length: C (mm)	Fly Ash Mixing Amount: D (%)
1	0.30	0.1	6	20
2	0.32	0.2	12	25
3	0.34	0.3	18	30

**Table 12:** Orthogonal test table L9 (3^4^).

Test Number	W/B	Fiber Volume Fraction (%)	Fiber Length (mm)	Fly Ash Mixing Amount (%)
	A	B	C	D
BF-1	1 (0.30)	1 (0.1)	1 (6)	1 (20)
BF-2	1 (0.30)	2 (0.2)	2 (12)	2 (25)
BF-3	1 (0.30)	3 (0.3)	3 (18)	3 (30)
BF-4	2 (0.32)	1 (0.1)	2 (12)	3 (30)
BF-5	2 (0.32)	2 (0.2)	3 (18)	1 (20)
BF-6	2 (0.32)	3 (0.3)	1 (6)	2 (25)
BF-7	3 (0.34)	1 (0.1)	3 (18)	2 (25)
BF-8	3 (0.34)	2 (0.2)	1 (6)	3 (30)
BF-9	3 (0.34)	3 (0.3)	2 (12)	1 (20)

**Table 13:** Overview of test specimens.

Number	Test Name	Specimen Size (mm^3^)	Number of Groups	Number/Group	Number of Specimens (Pieces)
1	Compressive strength	100 × 100 × 100	9	3	9 × 3 = 27
2	Splitting tensile strength	100 × 100 × 100	9	3	9 × 3 = 27
3	Flexural strength	100 × 100 × 400	9	3	9 × 3 = 27
4	Early crack resistance	800 × 600 × 100	6	1	6 × 1 = 6

**Table 14:** The 28 d compressive strength of the basalt fiber self-compacting high-strength concrete.

Number	A W/B	Fiber Volume Fraction: B (%)	Fiber Length: C (mm)	Fly Ash Mixing Amount: D (%)	Compressive Strength (MPa)
BF-1	1 (0.30)	1 (0.1)	1 (6)	1 (20)	55.10
BF-2	1 (0.30)	2 (0.2)	2 (12)	2 (25)	58.20
BF-3	1 (0.30)	3 (0.3)	3 (18)	3 (30)	57.60
BF-4	2 (0.32)	1 (0.1)	2 (12)	3 (30)	54.30
BF-5	2 (0.32)	2 (0.2)	3 (18)	1 (20)	54.70
BF-6	2 (0.32)	3 (0.3)	1 (6)	2 (25)	53.10
BF-7	3 (0.34)	1 (0.1)	3 (18)	2 (25)	51.90
BF-8	3 (0.34)	2 (0.2)	1 (6)	3 (30)	52.80
BF-9	3 (0.34)	3 (0.3)	2 (12)	1 (20)	52.50

**Table 15:** Extreme range of compressive strength.

Items	A W/B	Fiber Volume Fraction: B (%)	Fiber Length: C (mm)	Fly Ash Mixing Amount: D (%)
K1	170.9	161.3	161.0	162.3
K2	162.1	165.7	165.0	163.2
K3	157.2	163.2	164.2	164.7
K1^-^	56.70	53.77	53.67	54.10
K2^-^	54.03	55.23	55.00	54.40
K3^-^	52.40	54.40	54.73	54.90
R	4.30	1.46	1.33	0.80

**Table 16:** The 28 d split tensile strength of the basalt fiber self-compacting high-strength concrete.

Number	A W/B	Fiber Volume Fraction: B (%)	Fiber Length: C (mm)	Fly Ash Mixing Amount: D (%)	Splitting Tensile Strength MPa
BF-1	1 (0.30)	1 (0.1)	1 (6)	1 (20)	3.36
BF-2	1 (0.30)	2 (0.2)	2 (12)	2 (25)	4.45
BF-3	1 (0.30)	3 (0.3)	3 (18)	3 (30)	4.25
BF-4	2 (0.32)	1 (0.1)	2 (12)	3 (30)	3.92
BF-5	2 (0.32)	2 (0.2)	3 (18)	1 (20)	4.18
BF-6	2 (0.32)	3 (0.3)	1 (6)	2 (25)	3.64
BF-7	3 (0.34)	1 (0.1)	3 (18)	2 (25)	3.80
BF-8	3 (0.34)	2 (0.2)	1 (6)	3 (30)	3.59
BF-9	3 (0.34)	3 (0.3)	2 (12)	1 (20)	4.31

**Table 17:** Extreme range of the split tensile strength.

Items	A W/B	Fiber Volume Fraction: B (%)	Fiber Length: C (mm)	Fly Ash Mixing Amount: D (%)
K1	12.06	11.07	10.59	11.85
K2	11.73	12.22	12.69	11.88
K3	11.70	12.20	12.23	11.76
K1^-^	4.02	3.69	3.53	3.95
K2^-^	3.91	4.06	4.23	3.96
K3^-^	3.90	4.07	4.08	3.92
R	0.12	0.38	0.7	0.04

**Table 18:** The 28 d flexural strength of basalt fiber self-compacting high-strength concrete.

Number	A W/B	Fiber Volume Fraction: B (%)	Fiber Length: C (mm)	Fly Ash Mixing Amount: D (%)	Splitting Tensile Strength MPa
BF-1	1 (0.30)	1 (0.1)	1 (6)	1 (20)	4.67
BF-2	1 (0.30)	2 (0.2)	2 (12)	2 (25)	5.48
BF-3	1 (0.30)	3 (0.3)	3 (18)	3 (30)	5.55
BF-4	2 (0.32)	1 (0.1)	2 (12)	3 (30)	5.45
BF-5	2 (0.32)	2 (0.2)	3 (18)	1 (20)	5.68
BF-6	2 (0.32)	3 (0.3)	1 (6)	2 (25)	5.27
BF-7	3 (0.34)	1 (0.1)	3 (18)	2 (25)	4.95
BF-8	3 (0.34)	2 (0.2)	1 (6)	3 (30)	5.16
BF-9	3 (0.34)	3 (0.3)	2 (12)	1 (20)	5.31

**Table 19:** Extreme range of the flexural strength.

Items	A W/B	Fiber Volume Fraction: B (%)	Fiber Length: C (mm)	Fly Ash Mixing Amount: D (%)
K1	15.69	15.06	15.09	15.66
K2	16.41	16.32	16.23	15.69
K3	15.42	16.14	16.17	16.17
K1^-^	5.23	5.02	5.03	5.22
K2^-^	5.47	5.44	5.41	5.23
K3^-^	5.14	5.38	5.39	5.39
R	0.33	0.42	0.38	0.17

**Table 20:** Efficiency coefficient of concrete performance.

Number	Factor Combination	Assessment Indicators			Efficacy Coefficient			Total Efficacy Coefficient
		Compressive Strength (MPa)	Splitting Tensile Strength (MPa)	Flexural Strength (MPa)	d1	d2	d3	d
BF-1	A_1_B_1_C_1_D_1_	55.10	3.36	4.67	0.945	0.755	0.822	0.669
BF-2	A_1_B_2_C_2_D_2_	58.20	4.45	5.48	1.000	1.000	0.965	0.988
BF-3	A_1_B_3_C_3_D_3_	57.60	4.25	5.55	0.988	0.955	0.977	0.936
BF-4	A_2_B_1_C_2_D_3_	54.30	3.92	5.45	0.930	0.881	0.960	0.808
BF-5	A_2_B_2_C_3_D_1_	54.70	4.18	5.68	0.938	0.939	1.000	0.881
BF-6	A_2_B_3_C_1_D_2_	53.10	3.64	5.27	0.911	0.818	0.928	0.727
BF-7	A_3_B_1_C_3_D_2_	51.90	3.80	4.95	0.890	0.854	0.871	0.726
BF-8	A_3_B_2_C_1_D_3_	52.80	3.59	5.16	0.906	0.807	0.908	0.708
BF-9	A_3_B_3_C_2_D_1_	52.50	4.31	5.31	0.901	0.969	0.935	0.853

**Table 21:** Extreme range analysis of the total efficacy coefficient.

Items	A W/B	Fiber Volume Fraction: B (%)	Fiber Length: C (mm)	Fly Ash Mixing Amount: D (%)
K_1_^−^	0.864	0.734	0.701	0.801
K_2_^−^	0.805	0.859	0.883	0.814
K_3_^−^	0.762	0.839	0.848	0.817
R	0.102	0.125	0.182	0.016

**Table 22:** Crack length and maximum crack width of each cutting edge.

Test Piece Number	Crack Length/Maximum Crack Width for Each Blade Number (mm)						
	1	2	3	4	5	6	7
NC	596/0.3	362/0.27	622/0.28	502/0.3	583/0.32	317/0.28	565/0.32
K-0.1-12	525/0.25	-	346/0.2	453/0.28	533/0.2	-	418/0.22
K-0.2-6	422/0.25	350/0.18	328/0.2	-	302/0.15	-	450/0.2
K-0.2-12	262/0.1	-	319/0.18	-	324/0.2	145/0.18	-
K-0.2-18	-	292/0.18	-	158/0.16	-	-	-
K-0.3-12	-	-	185/0.12	-	125/0.06	-	-

**Note:** NC is the reference concrete specimen. K-0.1-12 represents a concrete specimen with a fiber volume fraction of 0.1% and a length of 12 mm. The blade numbers are 1–7 from left to right.

**Table 23:** Early evaluation index of the crack resistance.

Test Piece Number	Early Crack Resistance Performance Evaluation Indicators				
	n/piece	a/mm^2^/piece	b/piece/m^2^	c/mm^2^/m^2^	*η*
NC	7	75.53	14.58	1101.23	0
K-0.1–12	5	52.95	10.42	551.74	0.499
K-0.2–6	5	43.66	10.42	454.96	0.587
K-0.2–12	4	21.82	8.33	181.76	0.835
K-0.2–18	2	19.39	4.17	80.86	0.927
K-0.3–12	2	7.43	4.17	30.98	0.972

## Data Availability

The data used to support the findings of this study are available from the corresponding author upon request.
